# An Exposition of the Nature, Treatment, and Prevention of Continued Fever

**Published:** 1835-04-01

**Authors:** 


*An Exposition of the Nature, Treatment, and Prevention of Continued Fever.* By Henry M'Cormac, M.D.—London, 1835. 8vo. pp. 202.

If it be a humiliating consideration that the medical doctrines most influential in one age melt away before the increasing information of that which succeeds it, we may console ourselves with the reflection that every hypothesis engages its defenders in the accumulation of facts, which remain when the mist of false reasoning through which they were viewed has dispersed: the foundations of reasoning are thus confirmed and enlarged, while the experience of past error favours in some degree the progress of truth, by warning us from devious paths. Hence there are many subjects which we are much better qualified to investigate than our forefathers, though we may not as yet have arrived at much more accurate knowledge. These truths are illustrated by the progress of opinion on the subject of fever. Medical philosophers of all ages have been much occupied with endeavours to discover in what the essence of this disease consists; and it must be confessed that no more of the matter is positively known now than at the commencement of the inquiry: many important facts, however, have been brought to light; the effects of the still latent cause have been successfully elucidated, the most frequent sources of danger detected, and the disease thereby brought much more under the control of art.

For a long time the theory of fever was obscured by the substitution of chimeras for facts; at a more advanced period of the inquiry, hypotheses, not in themselves improbable, were made beds of Procrustes, on which unaccommodating facts were stretched or curtailed into conformity: in the present day, when facts are more accurately observed, and more diligently recorded, the propensity to undue generalization too frequently converts the means of knowledge into sources of error.

The opinions of Stahl and Hoffman, as modified and expanded by Cullen, may be considered as the first plausible explanation of the phenomena of fever. It is true that Cullen embarrassed his reasoning by his favourite hypothesis of spasm, which frequently made him lose sight of facts, or misconstrue them: notwithstanding, however, the senseless vituperation that has been bestowed upon this great physician by certain writers, who, when compared with him, "seem scarce so gross as beetles," it will appear, on candid consideration, that his views, on the whole, are infinitely less repugnant to facts than any of the exclusive hypotheses lately so much in vogue. Let it not be forgotten that Cullen makes the essence of fever to consist in the operation of some cause exerting a sedative influence on the nervous centres; a doctrine which, to this hour, has gained strength by the accumulation of facts, and which, though we have no absolute proof of its truth, bids fair to be ultimately established by the method of exclusion,—by the entire insufficiency or manifest falsehood of all other suppositions.

If asked to assign our reasons for believing fever to arise primarily from a sedative impression on the nervous system, we should give the following as the principal:

1. The universality of symptoms indicative of nervous depression, and the variability of all other symptoms. We see fevers with and without local inflammation; we see occasionally every variety in the state of the vascular system, and the functions immediately dependent upon it: but we never yet saw or heard of a case of regular continued fever, which was unattended at its commencement, and throughout its course, with manifest indications of diminished nervous power.

2. The general derangement of all the animal and vital functions, arguing an affection of that system whose influence is most universal over all the functions of life, namely, the nervous system.

3. The affinity between the first, that is, the essential stage of fever and those states of the system which are almost universally ascribed to a sudden depression of the nervous power; as in the instance of overwhelming affections of the mind. In the pallid aspect, cold surface, feeble pulse, oppressed respiration, and staggering gait of one whose mind has received a great and sudden shock, who can fail to recognize phenomena strikingly similar, nay almost identical, with those produced by the sudden invasion of fever?

The influence of those recent doctrines which refer all the phenomena of fever to some local inflammation, seems to be already on the wane. It is fortunate for the cause of truth that these exclusive hypotheses have all asserted their claim to attention nearly at the same time, and thereby tended to neutralize each other.

Dr. A. has spent half his life in dissecting people who died of fever: their brains were all inflamed: fever is *phrenitis.* Dr. B. has been equally indefatigable: there was nothing the matter with the patients' brains: they died of inflammation: fever is *enteritis.* Dr. C. has inspected the brains and intestines of the defunct with microscopic accuracy, and finds not in these the cause of death; but, lo! the lining membrane of the arteries was as red as scarlet: fever is *arteritis.*

Now, as our reason tells us that *all* these conflicting statements cannot be true, so the unprejudiced use of our eyes will convince us that no one of them is true. Whoever has been in the habit of dissecting the bodies of those who have died of fever, without having his mind preoccupied by an hypothesis, must be perfectly satisfied that, although some organ or organs are frequently, nay generally, found to have been inflamed, that no one of them is uniformly so; and that fatal cases of fever sometimes occur, in which morbid anatomy cannot detect any local appearance of the smallest importance.

Of all the inflammatory hypotheses, we believe that which makes the gastro-enteric mucous membrane the seat of fever to be by far the least objectionable, in a practical point of view; because, although inflammation be not the cause of fever, it is a very frequent complication: much more frequent, according to our own experience, than any other local affection.

An ingenious writer, Dr. Mackintosh, has observed that certain causes, which are partial in their operation, may have tended to mislead pathologists in the investigation of fever: thus, if Dr. Clutterbuck has found nothing but inflamed brains, "it must be kept in view that he practises in the greatest commercial city in the universe, among a people whose minds are, generally speaking, more actively employed than their bodies,—who are exposed to intense anxieties occasioned by extensive speculations and reverses of fortune,—who are either in a state of considerable mental excitement or depression. If to these considerations we add the effects of heavy meals and sedentary habits, impeding the functions of the stomach and bowels, it will be seen that there may be considerable foundation for the opinions this gentleman has been led to advance."

Again, with reference to the doctrine of Broussais, he observes, "In France it is no wonder that Broussais should so frequently find the mucous membrane of the stomach and intestines altered, both in appearance and structure, if the habits and modes of living of the people are recollected. The stewed meats, salads, oils, and sweets, consumed by Frenchmen among the higher ranks, together with the hard beer and acid wines which they drink, and the unwholesome food eaten by the lower ranks, all tend to produce irritation in the digestive organs. Sooner or later these irritating matters produce increased vascularity, which must frequently terminate in inflammation and ulceration." (*Practice of Physic*, vol. i., p. 32.)

These are philosophical remarks, and highly worthy of attention.

If it be erroneous to connect with the essence of fever any of those forms of inflammation, some one of which there is much reason to believe has always accompanied a large proportion of cases of this disease, it is still more erroneous to assign an undue importance to those more partial organic lesions which are observed to accompany particular epidemics, or, at most, to distinguish the fevers of one age or country from those of another. The most prevalent delusion of this kind (for such, we fear, it must be called,) in the present day, is that which connects fever necessarily with disease of Peyer's and Brunner's glands.

We by no means intend to question the accuracy of observation of several distinguished physicians who have noticed the appearance alluded to; but the silence on this point of a host of inquirers, equally diligent, and no less worthy of confidence, affords much reason to doubt the alleged uniformity of its occurrence. How is it that Broussais, and his numerous followers, whose peculiar opinions have led them to so frequent and scrutinizing an examination of the gastro-enteric mucous membrane, in cases of fever, can have been blind to so constant and obvious a phenomenon?

Is it not hence probable that this, like many other complications of fever, forms no essential part of the disease, but is confined to particular epidemics, periods, and localities?

This view of the subject is countenanced by a retrospect of the history of fever.

We find the most accurate writers of past centuries describing, as characteristic of fevers which fell under their observation, symptoms which are no longer to be found in those of our own time. Where, for instance, is now the putrid fever of this country, so graphically delineated by Huxham, in which the worst symptoms of scurvy seem to have been superadded to those of fever, and to have produced that dissolved and putrescent state of the fluids from which the fever derives its name, and in which it was then thought mainly to consist? No such cases now present themselves, though we occasionally observe a slight approximation to them; yet nobody doubts that the putrid fever of Huxham was essentially the same malady as the typhus of the present day.

Let those who would inquire philosophically into the pathology of fever rest assured that the subject can only be successfully elucidated by a comparison of many series of careful observations, made by different individuals, in different countries, and at distant intervals of time.

We have been waiting for an opportunity of introducing our author, Dr. M'Cormac; and, if we have been tardy in so doing, it is because his work, though on the whole an excellent one, labours under a defect of arrangement, which renders it difficult to know where to commence a notice of it. The whole book is one chapter; a peculiarity not to be commended in a treatise, the complexity of whose subject renders subdivision essential to perspicuity. The following is a judicious summary of the morbid anatomy of typhus fever,—that is, of continued fever in general; for our author is not disposed to admit the validity of nosological distinctions in this disease; wherein we entirely agree with him.

"After death, the brain is sometimes found slightly injected, and occasionally pale; in other cases, it may be unusually hard or soft; but frequently nothing peculiar is to be observed. The veins and sinuses are occasionally congested with blood. The membranes are sometimes slightly injected, and there is in many cases a greater or less effusion of serum; but this is a common circumstance after death, as all who are engaged in anatomical investigations are aware. In rare cases, a little pus or sero-purulent fluid is discovered; sometimes there is a gelatinous effusion, and at others partial adhesions of the membranes. So much, concisely speaking, for the changes presented after death by the brain. Of the spinal marrow, nothing particular is on record. As for the fluid seen flowing out, it is a matter of almost universal occurrence after death, being, in fact, a normal condition. The details respecting the apparatus of the sympathetic nerves are still less satisfactory. Andral has seen the semilunar ganglions red, but does not venture to assert that it was a mark of disease; and I fully agree with this writer, when he affirms that the state of the nervous centres after death does not, in the great majority of cases, account for their functional derangement during life. We have been stuffed to satiety with the specious details (in too many cases really anything but satisfactory,) of morbid anatomy; and it is refreshing when such a first-rate pathologist assigns them their just value. In truth, the appearances after death in the brain will not, as has often been asserted, explain the phenomena of disease, any more than they will those of life. The language of morbid anatomy is not too definite; and why relate too minutely the details of phenomena, which, however important, must be witnessed, to be known and appreciated? The morbid varieties observed in the respiratory organs, though highly important, are not very numerous: they are, however, frequently observed, and constitute, along with other alterations, a frequent cause of death. They consist of the ordinary results of inflammation and congestion. The bronchial lining is more or less red, accompanied with a considerable effusion of mucus. Armstrong affirms that typhus is always attended with a special bronchitis; indeed, he insists so much on it, that we might almost conclude that he looks upon it as the leading cause of typhus fever: other pathologists certainly do not support him in the lengths to which he has gone in this respect. The lungs are frequently as if gorged with blood or serum; and hepatization, the result of pneumonia, is not an uncommon result. I have often seen it, more or less extensive, in the lungs of those who died of fever. Andral speaks of a lesion, frequently to be met with, in which the pulmonary parenchyma is injected with blood, as in hepatization, but more like softened spleen. The cavity, as it is called, of the pleura, in some fatal cases, contains a reddish effusion: it does not appear that this is any consequence of inflammation; the pleura, besides, seldom exhibits any evidence of it. The pericardium occasionally presents a serous effusion of a ruddy aspect; and the same may be remarked of the peritoneum. The liver is not often organically affected in fever; and, when it is so, we may perhaps conclude that it is seldom connected with the disease. As to the bile, it is sometimes altered as to quality and quantity; but we can hardly connect any of the phenomena of the disease with its appearances, or those of the liver; a few rare cases excepted. The urinary apparatus seldom presents organic lesion. The spleen is not rarely soft and voluminous; more frequently, Andral remarks, than in any other disease; and Louis also is of opinion that this viscus is more frequently affected than any other in fever. The heart seldom presents any lesion: sometimes, however, its internal parietes, and those of the large vessels, are redder than usual; but no peculiar conclusion can be drawn from these circumstances. Little need be said in this place respecting the blood; its alterations have been already spoken of. The extreme liquidity and other changes sometimes presented by this vital fluid are not peculiar to fever.

" The alterations which occur in the intestinal canal have received a large share of attention during late years, particularly from the strenuous attempts which have been made to localize the seat of fever in this viscus; and which attempts are consequently based upon the affirmation of the universal presence of local affection. It is quite certain, however, as appears from the avowal of the best pathologists, that very many cases of the disease above mentioned occur without the slightest perceivable organic alteration, adequate to account for them. If such a circumstance prove that fever is not owing to a gastro-enteritis, it also shows as strongly, in my opinion, that a follicular exanthem, so styled, is not the cause either. It has been said, however, that, as erysipelas is sometimes found to leave little or no trace on the skin after death, so may the organic evidence of inflammation of the mucous membrane also disappear in like circumstances. I do not think that great attention is to be attached to this explanation, though it should not be overlooked: great consideration, nevertheless, is due to those who pointed out the frequent and too-generally neglected lesions of the intestinal canal, such as they occur in fever. The great object is to obtain as large a list of the functional and organic phenomena as possible; but to assign the source, and to demonstrate the order of causation in the disease, is, I fear, as yet beyond our grasp; if it may ever prove otherwise. The transient appearances produced after death by the passage of blood or bile, or the traces of the incipient dissolution of the frame, are hardly worthy of notice, except to avoid confounding them with the changes occurring in life. The mucous membrane is occasionally here and there more or less injected; it sometimes also undergoes the change which is called *ramollisement*, or softening. On the whole, the organic changes of the stomach are not considerable in fever. The small intestines, especially towards the end of the ileum, as I have said before, present frequent traces of disease. Of these, the most remarkable is that peculiar affection, or exanthem, as it is styled, of the follicles, which has received the name of *dothinenteritis.* The agminated and the isolated crypts or follicles, or those of Peyer and Brunner, as they are sometimes called, may both prove the subjects of this phenomenon: the first, however, are more frequently affected than the latter. When they have undergone this morbid change, they rise above the surface of the surrounding mucous membrane, than which they are sometimes softer, and sometimes harder; the mucous membrane itself may or may not be otherwise affected. Occasionally they occur in the cæcum in plates, as in the ileum, but isolated in the rest of the large intestines. Sometimes the diseased follicles may be traced individually in the plates; at others, they are indistinguishable from each other. They may proceed to resolution, or they may form eschars, which, falling off, display ulcers, varying in extent, from the size of a single follicle to that of a whole plate: in the former case they are often exactly round. These ulcers sometimes destroy the mucous membrane, to the extent of many inches, not only in the ileum, but in the cæcum; at other times they are few and small. The ulcers may cicatrize if the patient survive: in a few cases, however, they lead to perforations, which, permitting the effusion of the contents of the intestines into the cavity of the peritoneum, cause inflammation and death. This result does not occur except in advanced stages of the disease. The exanthem, if we may call it so, does not present the regular phases seen in small-pox: it may exist during the greater portion of the disease, and even after the fever has terminated. After what I have said, I need hardly repeat again that, together with its results, it is only an occasional contingency in fever, and seemingly more frequent on the continent than in this country; from what cause, however, if it be thus, I do not pretend to say. They are rarely seen in old men: these, however, when seized with fever, present the same general symptoms with young persons in whom this phenomenon occurs. Independent, however, of the unquestionable fact that this exanthem, as some call it, is far from invariable in fever, those who most warmly contend for its being the cause of the disease admit that it has been detected in phthisis and scarlatina. The mesenteric glands always undergo a morbid alteration, corresponding to the severity of the exanthem. They enlarge in size, grow soft and discoloured, and sometimes contain pus. As to the contents of the intestinal tube, a collection of gas, occasionally amounting to meteorism, is sometimes seen in bad fevers; worms are a matter of common observation, also vitiated bile and blood." (P. 83.)

Admitting the general prevalence of inflammatory action in some organ or other, though not uniformly in any one, the cause of such inflammatory action becomes an interesting object of inquiry. It may be offered in explanation, that the functions of the whole vascular system being deranged, those organs suffer most which are most predisposed to disease; but it is to be objected to this hypothesis, first, that local inflammation is frequently found to accompany fever, where the general affection of the vascular system is not of a kind at all likely to induce this morbid state; where the actions of the arterial system are enfeebled and diminished. Secondly, that it is not warrantable to assume that any organ was predisposed to disease, in cases where the patient was apparently in perfect health immediately previous to the accession of fever, which is very often the fact.

In attempting an hypothetical solution of what is confessedly beyond our knowledge, we should not lose sight of the valuable and original observation of Dr. Wilson Philip,* that the division of the nerves of a vital organ immediately deranges the action of its vessels: of course, defective innervation, from whatever cause, may be expected to produce some degree of the same effect. Now, if the essence of fever be supposed to consist in the action of a depressing cause on the nervous centres, it may easily be conceived that such cause operates more energetically on one portion of the nervous mass than another, and hence that vascular derangement will be most conspicuous in parts whose nerves are derived from those portions of the nervous mass which are more immediately under the influence of the depressing cause. In accordance with this view of the subject, Dr. M'Cormac makes the following remarks.

"So far as the process of innervation is concerned in the various functions of the animal economy, in so far will these functions become liable to derangement, when the seat of innervation itself—the brain, spinal marrow, and their dependencies, is subjected to functional or organic disease. But, as we do not know how far these are connected in health, it is at present, *à fortiori*, impossible to pronounce with precision upon their relations in disease. It is a truly remarkable circumstance that, in fever, inflammation will supervene in various viscera, at a period when no one would suppose that sufficient activity of the circulation did not exist for the purpose. It is more than probable that, independent of the functional lesions liable to be occasioned by the advent of vitiated blood in the brain, a proneness to various organic alterations will thereby be produced." (P. 73.)

Another point of some interest is the nature or kind of the inflammatory action which occurs in fever. Is it common inflammation, or is it specific? One might almost be led to conclude that there was something specific in its character, from the greater tendency to spontaneous resolution which it exhibits than ordinary inflammation occurring in the same

* Philosophical Transactions for 1827.

textures: thus we frequently observe, in the progress of continued fever, a very acute degree of inflammation lighted up in an important organ,—in the brain, for instance; we find that such inflammation continues, in spite of our attempts to remove it, for a length of time, during which ordinary inflammation could hardly be expected to exist, without fatal lesion of the part; yet, the fever having run its course, convalescence ensues, and the patient escapes without any permanent injury of the organ. Our author reasons on the subject thus.

"It is said, and with much justice, that the inflammations in fever are of a different character from those which take place idiopathically, or without fever in the first instance. Certainly they will not always bear the same activity of treatment. But are we entitled to affirm that the inflammation which superinduces fever, perhaps eventually of a typhoid cast, is always different from the inflammation which often occurs in fever? I should think not. Many are of opinion that the alteration which takes place in the blood causes the inflammations which occur while it is in this state to assume a different character: certainly in so far there will be a difference; but is it ascertained that there is any other? Different German and English pathologists think that the blood, in consequence of not undergoing the usual changes, acquires a narcotic character; but, although I would agree with them as to the facts which this term is intended to indicate, I should prefer simply recounting them, without using it, as I consider that to assume unnecessarily the existence of an uncertainty is rather calculated to lead us into error. Assuredly the alterations which the blood undergoes, as also its decrease, (a fact which seems to be occasionally overlooked,) must occasion marked changes in the processes of innervation, if not a partial suspension of them. The excessive diminution of the mass of the blood, from wounds, venesection, and flooding, is known to cause convulsions, loss of vision, hearing, and other nervous phenomena: now, in the latter stages of fever, there is not only a great change in the constitution, but also, as I have just observed, a notable diminution in the quantity of the blood. The universal prostration, *lähmung*, as the Germans expressively term it, which occurs in that form of fever called typhus, must undoubtedly be mainly occasioned by the excessive vitiation of the vital fluid.

"It is wonderful how late pneumonia will occasionally ensue in fever: I have seen it take place on the twentieth day, and prove nearly fatal. It is unaccountable how such a process can be set up in such opposite conditions of the animal economy—robust health, the one extreme, and a state of the lowest and most miserable depression, the other. Andral, and many other observers, as well, have seen fatal pneumonia ensue during convalescence." (P. 75.)

The treatment of fever is very fully and judiciously discussed by our author. The subject is too extensive to be entered into in a review, and too familiar to admit of much new illustration. While the pathology of this disease has strikingly exemplified the fallacy of medical reasoning, the records of practice show with how much caution the testimony even of able and candid men is to be received with reference to facts supposed to be indisputable, and of general applicability. Cold affusion, if we are to believe Dr. Currie, seldom fails to cut short fever, if early applied. What is the result of more mature experience?—that its inefficacy has nearly banished the use of it from practice.

The same virtue has been attributed, on high authority, to bleeding; yet, if there be a fact in the treatment of fever now generally agreed on among practitioners, it is that the disease cannot, under ordinary circumstances, be cut short by any means whatsoever.

The treatment of fever, in the hands of a practitioner who is content with facts, and not addicted to any particular hypothesis, is sufficiently obvious. The division of fevers introduced by Dr. Armstrong, into *simple*, *inflammatory*, and *congestive*, is of little value in pathology, because every fever is, at its commencement, congestive, however short a time it may remain so; and every fever may in its course become inflammatory. This distinction, however, is of great utility in a practical point of view. When the morbid cause is so concentrated, or the powers of the system so inadequate to contend against it, that arterial reaction is not effectually established, the fever is called congestive. Here the distinct indication is, if possible, to rouse the arterial system into action, and thus convert the disease into one or other of its more tractable forms—the simple or the inflammatory. For this purpose we know of no means so effectual as the use of powerful emetics. While this class of medicines has been much too highly extolled as a means of cutting fever short, there is a more important use of them, which is less familiar to practitioners, that of rousing the circulation in fever attended with deficient arterial action: in such cases, the exhibition of a strong emetic will frequently change the aspect of the disease, and establish a salutary reaction. The practitioner need not be deterred from the use of such remedies by the apparent prostration of the vital powers: nauseating medicines indeed are by all means to be avoided; but the full effect of an emetic is always stimulating, and is useful in congestive fever, on the same principle as in malignant cholera. A large dose of ipecacuanha is perhaps the best emetic to use in such cases. Some other means, as the warm bath and gentle stimulants, are naturally indicated, and will be found serviceable in this form of fever.

Simple fever, where the system resists with sufficient vigour the impression of the morbid cause, and no local inflammation supervenes, is a case which may be consigned to the *vis medicatrix naturæ* with every prospect of a fortunate result: except, indeed, attention to the state of the bowels, ventilation, cleanliness, and diet, we know of no means which are not more likely to prove injurious than beneficial.

In the inflammatory form of fever, the means available for the subjugation of ordinary inflammation are indicated, but under certain limitations. It sometimes happens that the local affection may be subdued at the commencement, and the disease converted into simple fever, which must be left to run its course, for the best of all reasons—that we have no means of arresting it; but it much more frequently happens that the local inflammation cannot be entirely subdued, and the object of the practitioner should then be so to moderate it, as to prevent its terminating in organic lesion. It is here that rash antiphlogistic practice is so pernicious, and the bad effect of certain exclusive pathological views so conspicuous. The physician who, having detected inflammation of an important organ in a case of fever, makes up his mind that the essence of the disease consists therein, that such inflammation must at all events be entirely subdued, and that depletion must be persevered in till this end be attained, will in all probability fail in his immediate object, and, what is worse, will sink his patient into such a state of debility as to render recovery extremely doubtful. We repeat, that the practitioner who regards fever as simple inflammation of the brain, bowels, or any other part, and acts implicitly on this supposition, will destroy many patients, who, if left to nature, would have recovered: it is fortunate, however, that most of these inflammatory pathologists *do not* act implicitly on their own doctrine; thereby in effect admitting its invalidity.

The treatment of inflammation accompanying fever sometimes exercises severely the judgment of the practitioner. It may happen, on the one hand, that local inflammation continues so acute as to endanger disorganization of a vital part; and, on the other, that antiphlogistic means have already been pushed as far as prudence will allow. It is under these circumstances that mercury becomes so valuable an auxiliary, and frequently enables us to arrest the local disease, and to save the patient. Much more extensive utility has been ascribed to mercury in fever; but on this subject we entirely concur in the sentiments of our author.

"Mercury, independent of its purgative action, has been used, and frequently recommended, both as a prophylactic and therapeutic agent in fever. Many able physicians have given it as their opinion that, when it could be given so as to affect the mouth, the fever would cease. Here, however, we labour under the ambiguity as to whether the mercury acted because the fever ceased, or in spite of the fever, and so caused its resolution. The sum of the evidence on this subject seems to me to prove that sometimes the one occurrence took place, and sometimes the other; that some persons are capable of having their mouths affected by mercury in fever, which thereupon disappears; and that others, either from the stronger hold of the disease, or some inexplicable peculiarity of constitution, are insusceptible of the mercurial influence until the fever ceases. Yet patients will occasionally die of fever, notwithstanding the production of ptyalism: this, however, appears to be an exception to the general rule. From all, however, that I have been able to learn on the subject from others, as well as from my own experience, I have come to the conclusion that the administration of mercury as a sialagogue in fever, and with a view to cut short the complaint, though sometimes successful, is on the whole an uncertain remedy, and inferior in point of efficacy to other modes of medication. Of course, I do not include in this remark the exhibition of mercury with opium in fever complicated with inflammation, after bloodletting has been carried as far as it is practicable, nor when it is given in small doses as an alterative. I have myself, upon the recommendation of those who had used it, given mercury in several instances during simple fever, to an extent sufficient to have produced salivation in a person in health: this result, however, never occurred, either during the complaint or after it had ceased; nor did it produce any influence on its duration, or otherwise. I have never tried it in a very bad or fatal case; and it will be obvious, from the preceding, that I do not feel inclined, on the whole, to recommend mercury in the form of a sialagogue as a general remedy, in ordinary cases of fever." (P. 173.)

We could have wished to have illustrated the treatment of fever by frequent references to Dr. M'Cormac's book; but, as we have before observed, this treatise, however excellent, is only so in the aggregate—it would be very difficult to cull its anthology: we must therefore be content to recommend it strongly as an elaborate and judicious review of facts and opinions, and withal a philosophical and practically useful treatise on the important subject of fever. A little more attention to arrangement would have made it an excellent book of reference.

